# Author Correction: High-throughput screening of genetic and cellular drivers of syncytium formation induced by the spike protein of SARS-CoV-2

**DOI:** 10.1038/s41551-024-01177-8

**Published:** 2024-01-24

**Authors:** Charles W. F. Chan, Bei Wang, Lang Nan, Xiner Huang, Tianjiao Mao, Hoi Yee Chu, Cuiting Luo, Hin Chu, Gigi C. G. Choi, Ho Cheung Shum, Alan S. L. Wong

**Affiliations:** 1 Laboratory of Combinatorial Genetics and Synthetic Biology, School of Biomedical Sciences, The University of Hong Kong, Pokfulam, Hong Kong SAR China; 2 Centre for Oncology and Immunology, Hong Kong Science Park, Shatin, Hong Kong SAR China; 3 Department of Mechanical Engineering, The University of Hong Kong, Pokfulam, Hong Kong SAR China; 4 Advanced Biomedical Instrumentation Centre, Hong Kong Science Park, Shatin, Hong Kong SAR China; 5 State Key Laboratory of Emerging Infectious Diseases, Department of Microbiology, The University of Hong Kong, Pokfulam, Hong Kong SAR China; 6 Centre for Virology, Vaccinology and Therapeutics, Hong Kong Science Park, Shatin, Hong Kong SAR China; 7 Department of Infectious Disease and Microbiology, The University of Hong Kong-Shenzhen Hospital, Shenzhen, People’s Republic of China

**Keywords:** High-throughput screening, Mutagenesis, Pathogens, Synthetic biology, Mechanical engineering

Correction to: *Nature Biomedical Engineering* 10.1038/s41551-023-01140-z, published online 23 November 2023.

In the version of this article initially published, the name of Ho Cheung Shum was shown incorrectly (as Anderson H. C. Shum) and has been amended in the HTML and PDF versions of the article.

