# Supplementary material for: High-throughput screening of genetic and cellular drivers of syncytium formation induced by the spike protein of SARS-CoV-2
Source: Nat Biomed Eng. 2023 Nov 23;8(3):291–309. doi: 10.1038/s41551-023-01140-z (PMC10963270; doi:10.1038/s41551-023-01140-z)
Supplement: Supplementary file 1 — Supplementary figures and tables. [file 41551_2023_1140_MOESM1_ESM.pdf]

nature biomedical engineering

Article

https://doi.org/10.1038/s41551-023-01140-z

High-throughput screening of genetic and cellular drivers of syncytium formation induced by the spike protein of SARS-CoV-2

In the format provided by the authors and unedited

Contents

**Supplementary Fig. 1**	Library design and quality assessment for the Spike deep mutational scans
**Supplementary Fig. 2**	Mutability scores of all single mutations of the FPPR and furin cleavage site region of SARS-CoV-2 Spike
**Supplementary Fig. 3**	Assay ranges in defining fusion-(in)competent variants using droplet microfluidics-based and size-exclusion selection-based strategies
**Supplementary Fig. 4**	Testing a reverse selection approach to collect fusion-resistant cells
**Supplementary Fig. 5**	RNA-seq and gene ontology enrichment analysis on *AP2M1* and *FCHO2* knockout A549-ACE2-Cas9 cells
**Supplementary Fig. 6**	Cell viability after treatment with endocytosis inhibitors
**Supplementary Fig. 7**	CME inhibition does not affect Spike and ACE2 surface expressions and ACE2 binding
**Supplementary Fig. 8**	Clathrin-mediated endocytosis inhibition reduces SARS-CoV-2 virus RdRp level and Spike-pseudotyped virus susceptibility
**Supplementary Fig. 9**	Clathrin-mediated endocytosis inhibitors impede SARS-CoV-2 live virus-induced pathological damages in hamster lung tissues
**Supplementary Table 1**	Constructs used in this study
**Supplementary Table 2**	Primers used in this study

a

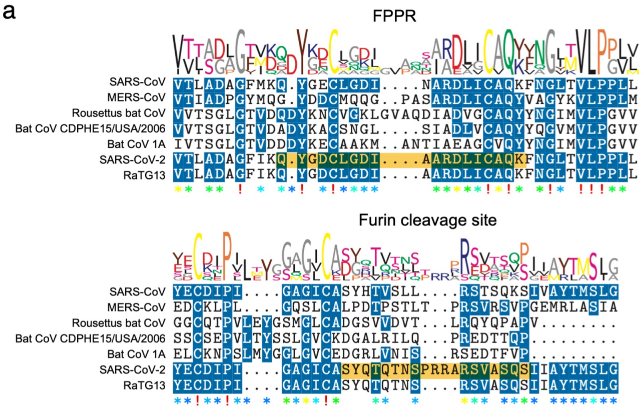

b

FPPR	Observed mutations	Furin cleavage site	Observed mutations
Q836	L,H,R,E,K	S673	T,G,I,R
Y837	H	Y674	F
G838	V,S,D	Q675	H,R,K,L
D839	Y,N,E,G,V,A	T676	I,N,S
C840		Q677	H,R,L,P,Y
L841	R,I,F	T678	I,N,A
G842	S,V,D,C	N679	K,I,S,Y,D
D843	G,N,Y	S680	F,Y,P,T
I844	V,S,T	P681	R,H,L,S,C,Y
A845	S,V,T	R682	L,W,Q
A846	S,V,T	R683	L,W,Q
R847	K,T,I	A684	V,T,S
D848	E	R685	L
L849	F,I	S686	G,N,I
I850	L,V,T,F	V687	L,I,A
C851		A688	V,T,S,G,P,D
A852	S,V,T	S689	I,N,G,R,T
Q853	L,R	Q690	H,R,L,K
K854	N,R,E,T	S691	F,P,A,Y

c

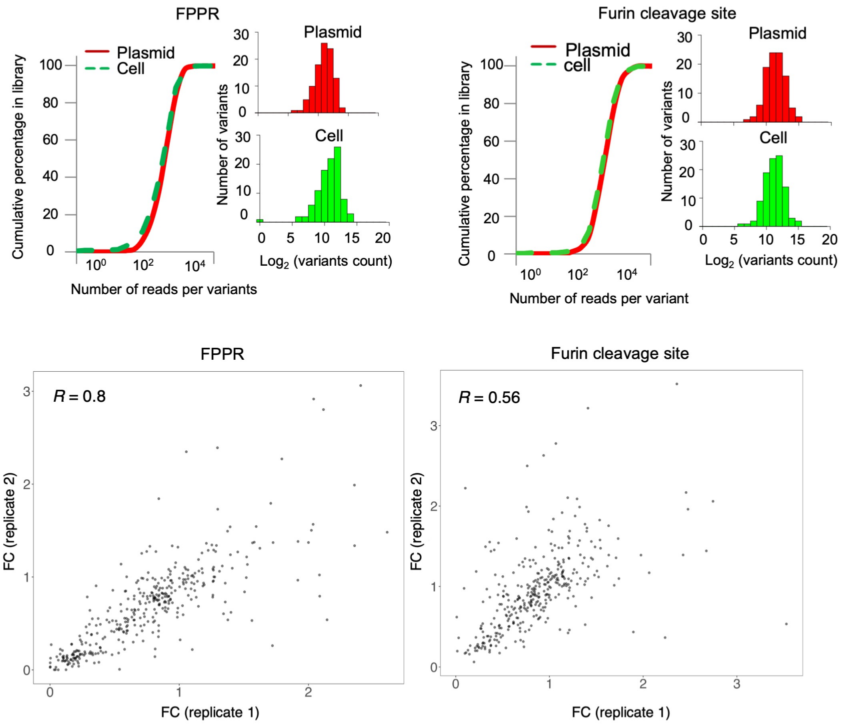

Supplementary Fig. 1 | Library design and quality assessment for the Spike deep mutational scans. a, Protein sequence alignment of SARS-CoV-2's FPPR and furin cleavage site regions with other coronaviruses. SARS-CoV-2 reference strain (GenBank accession number: YP_009724390.1), SARS-CoV (YP_009825051.1), MERS-CoV (YP_009047204.1), Rousettus bat CoV (YP_006908642.1), Bat CoV CDPHE15 (YP_008439202.1), Bat CoV 1A (YP_001718605.1), RaTG13 (MN996532). **b**, SARS-CoV-2 mutations at FPPR and furin cleavage site regions reported in the COG-UK-ME. **c**, Cumulative distribution of sequencing reads for the DMS library of Spike variants in the plasmid pools extracted from *E. coli* and the infected sender cell pools collected immediately before mixing with the receiver cells. High coverage of the FPPR library within the plasmid (100%) and infected cell (99.2%) pools, as well the Furin cleavage site region library within the plasmid (100%) and infected cell pool (99.4%), were detected. **d**, High reproducibility of profiling results was detected between two biological replicates in assessing the syncytium-forming potential of Spike variants at the FPPR and furin cleavage site region. The FC value represents the fold change comparing each variant's relative abundance in GFP-positive cell pool versus the cell pool before mixing and is normalized to wild-type. *R* is the Pearson correlation coefficient.

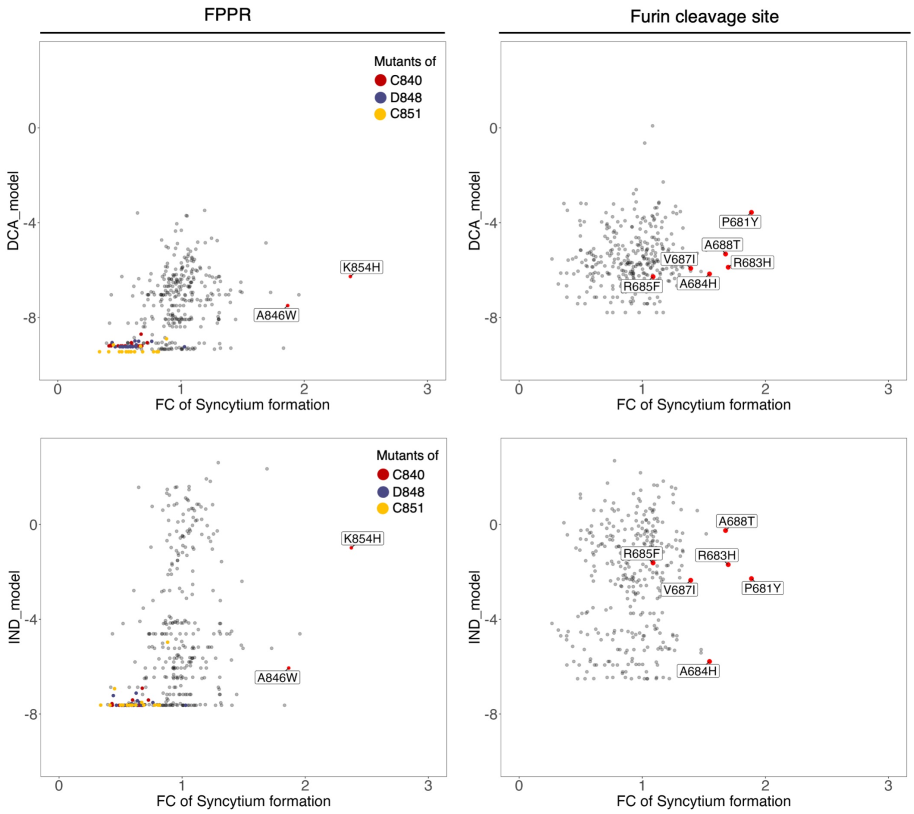

Supplementary Fig. 2 | Mutability scores of all single mutations of the FPPR and furin cleavage site region of SARS-CoV-2 Spike. The DCA and IND scores are plotted against the Fold Change (FC) obtained from the Spike DMS library profiling for each mutant. Mutants validated in this study are highlighted and labelled.

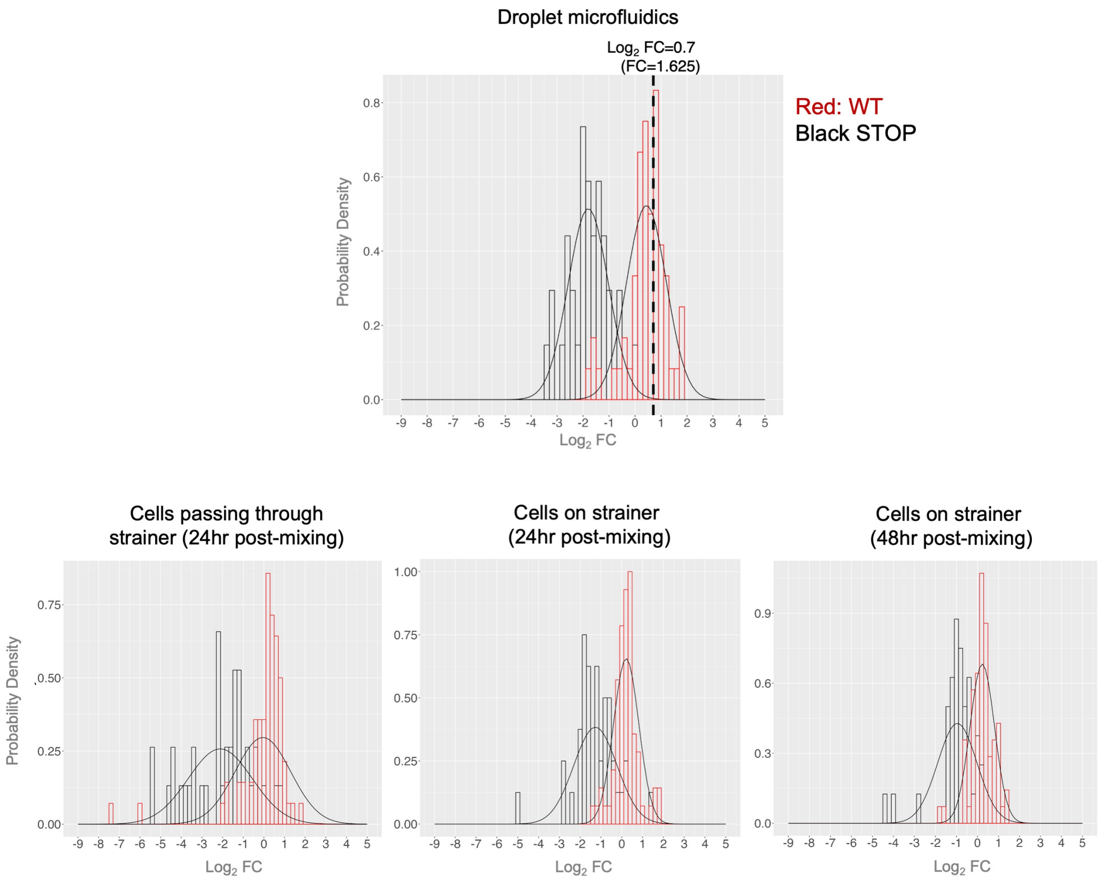

Supplementary Fig. 3 | Assay ranges in defining fusion-(in)competent variants using droplet microfluidics-based and size-exclusion selection-based strategies. Histograms showing the distribution of syncytium-forming potentials (measured by log2 Fold Change (FC)) of the WT Spike variants with synonymous codons and those with stop codons, assayed via the droplet microfluidics-based system and the size exclusion-based systems.

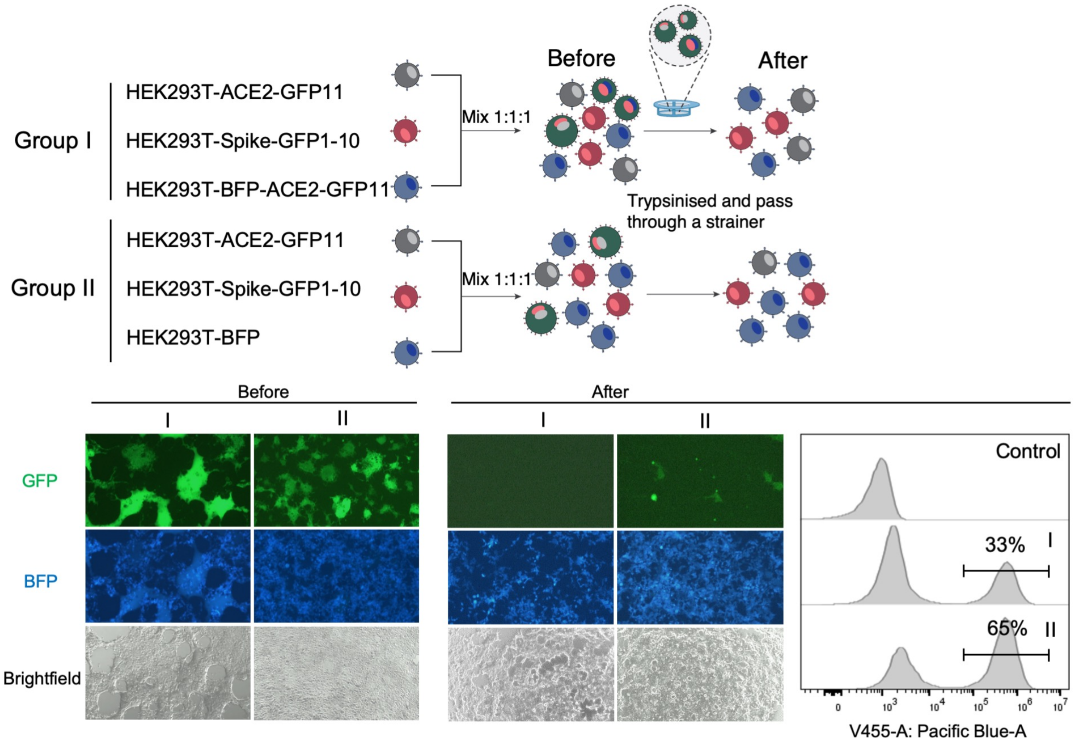

Supplementary Fig. 4 I Testing a reverse selection approach to collect fusion-resistant cells. HEK293T cells expressing ACE2-GFP11 and HEK293T cells expressing Spike-GFP1-10 were mixed with either HEK293T expressing BFP-ACE2-GFP11 or HEK293T expressing BFP only in 1:1:1 ratio. The cocultured cells were trypsinized and passed through a 40µm cell strainer to remove the fused cells. Cells were imaged before and after passing through the strainer, and representative images are shown. The percentage of BFP^{+} cells was measured by FACS.

a

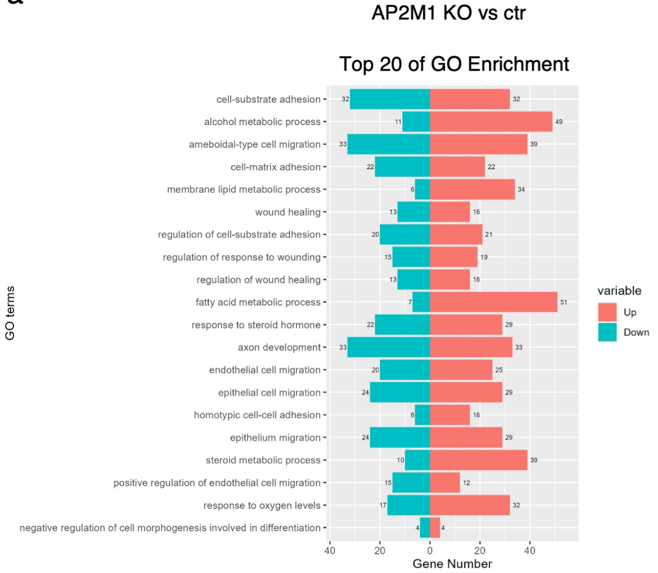

b

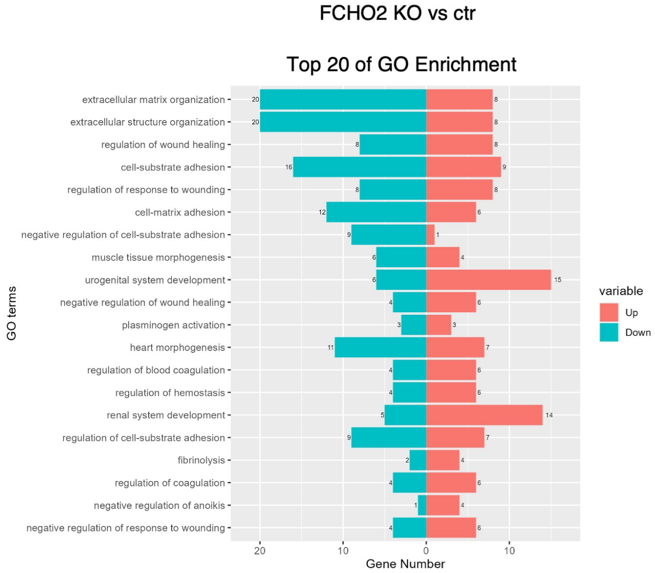

Supplementary Fig. 5 | RNA-seq and gene ontology enrichment analysis on *AP2M1* and *FCHO2* knockout A549-ACE2-Cas9 cells. a-b, Genes that are differentially expressed in *AP2M1* (a) and *FCHO2* (b) knockout (KO) cells when compared with control (ctr) cells were identified by RNA-seq analysis. Data were collected from three biological replicates. The up- and down-regulated genes were subjected to gene ontology (GO) enrichment analysis. The top 20 hits of biological processes in the GO enrichment analysis are shown.

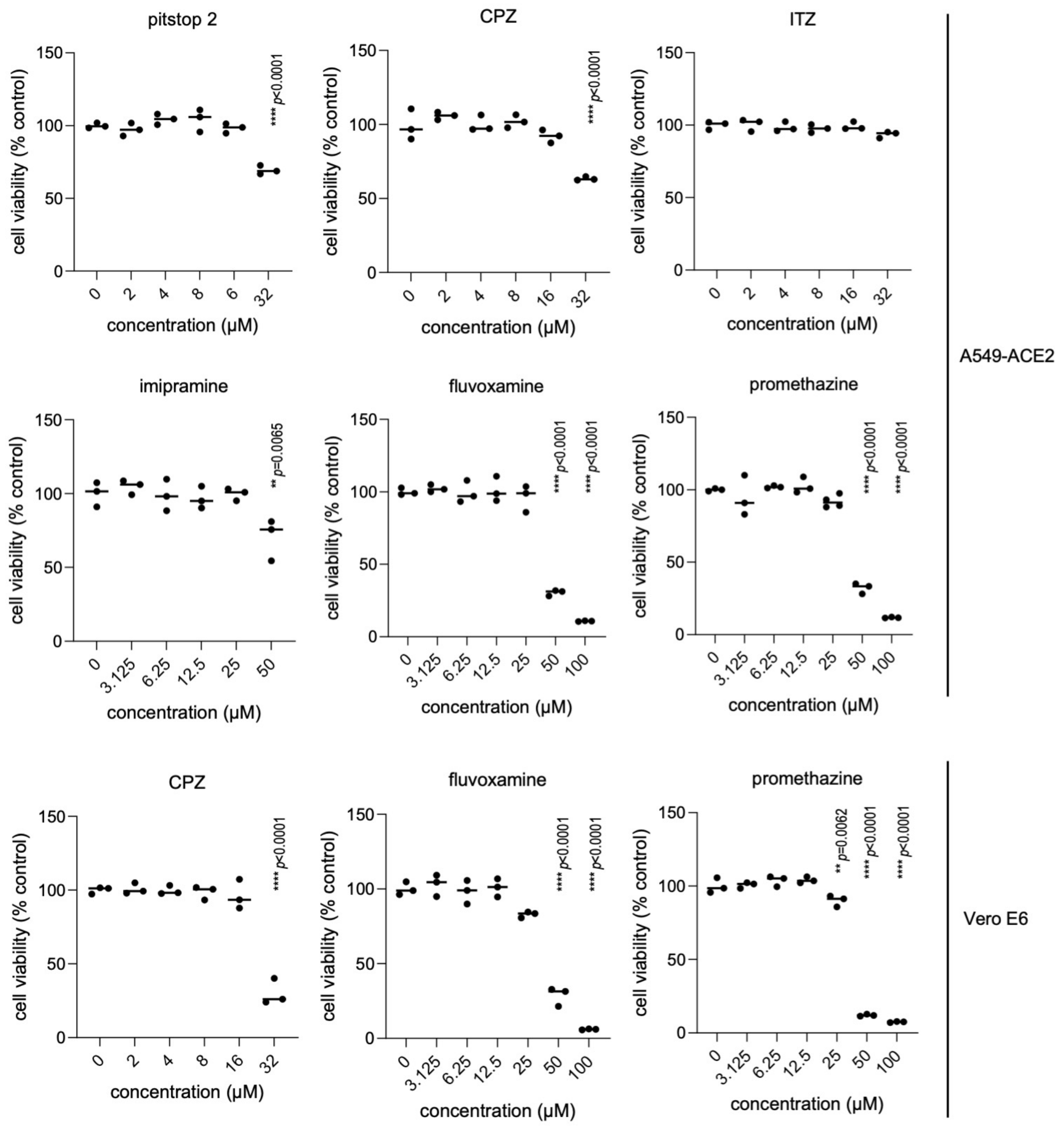

Supplementary Fig. 6 | Cell viability after treatment with endocytosis inhibitors. Viability of the drug-treated A549-ACE2 and Vero E6 cells was determined by XTT assay and is normalized to the DMSO-treated control. P-values indicated were compared with DMSO treated control. Statistical significance was determined using one-way ANOVA.

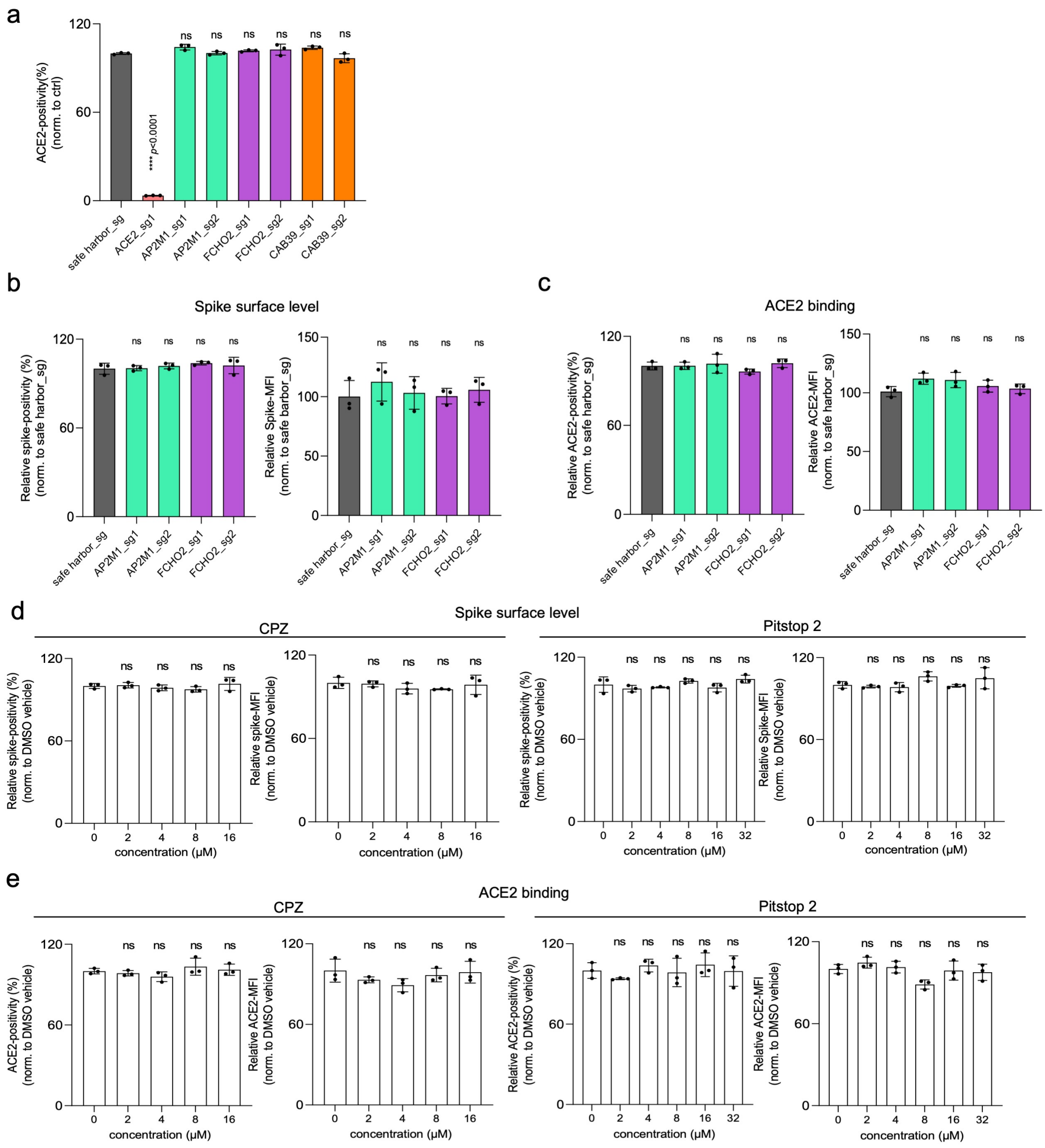

Supplementary Fig. 7 l CME inhibition does not affect Spike and ACE2 surface expressions and ACE2 binding. a, ACE2 surface expression in gene knockout A549-ACE2 cells. ACE2 surface staining was performed on the sgRNA-infected A549-ACE2-Cas9-BFP cells at 10 days post-infection, and the percentage of ACE2 positivity is normalized to that in the control cells infected with safe harbor-targeting sgRNA. **b-c**, Spike surface expression (b) and ACE2 binding (c) of *AP2M1* and *FCHO2* knockout HEK293T sender cells. **d-e**, Spike surface expression and ACE2 binding of drug-treated HEK293T sender cells, and the percentages of Spike and ACE2 positivity are normalized to that in the DMSO-treated control. Data shown are mean ± SD (n = 3), P-values indicated were compared with the safe harbor sgRNA controls (a-c) or DMSO treated control (d-e). ns represents no significant difference. Statistical significance was determined using one-way ANOVA.

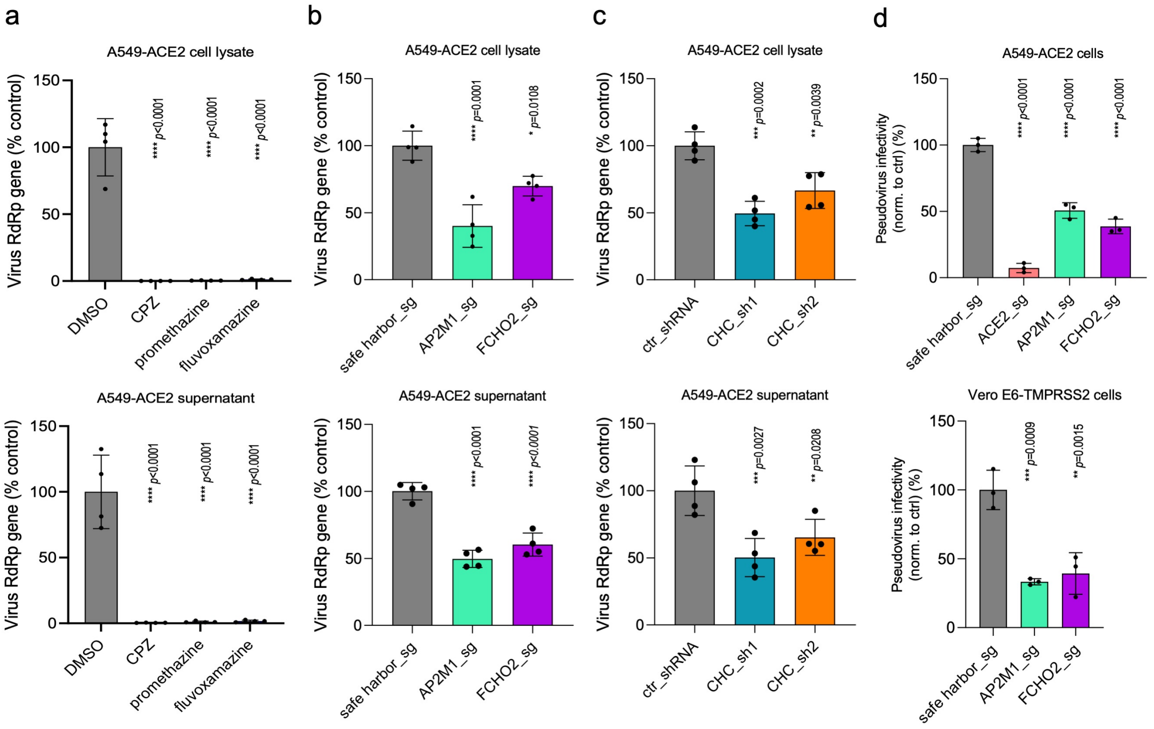

Supplementary Fig. 8 | Clathrin-mediated endocytosis inhibition reduces SARS-CoV-2 virus RdRp level and Spike-pseudotyped virus susceptibility. a-c, qRT-PCR measurements of SARS-CoV-2 RdRp levels in CME inhibitor-treated (a), *AP2M1* and *FCHO2* knockout (b), and *CHC* knockdown (c) A549-ACE2 cells. A549-ACE2-Cas9 cells were used in (b). The RdRp levels in both cell lysate and supernatant after SARS-CoV-2 D614G virus infection were measured. **d**, Spike-pseudotyped virus susceptibility test of *AP2M1* and *FCHO2* knockout A549-ACE2 and Vero E6-TMPRSS2 cells. The pseudovirus infectivity was determined by measuring the GFP^+ cells within the sgRNA-containing (BFP^+) A549-ACE2-Cas9 and Vero E6-TMPRSS2-Cas9 cell populations. Data shown are mean ± SD (n = 4 for (a-c); n = 3 for (d)). P-values indicated were compared with DMSO-treated control (a), safe harbour-targeting sgRNA (b, d), or control (ctr) shRNA (c). Statistical significance was determined using one-way ANOVA.

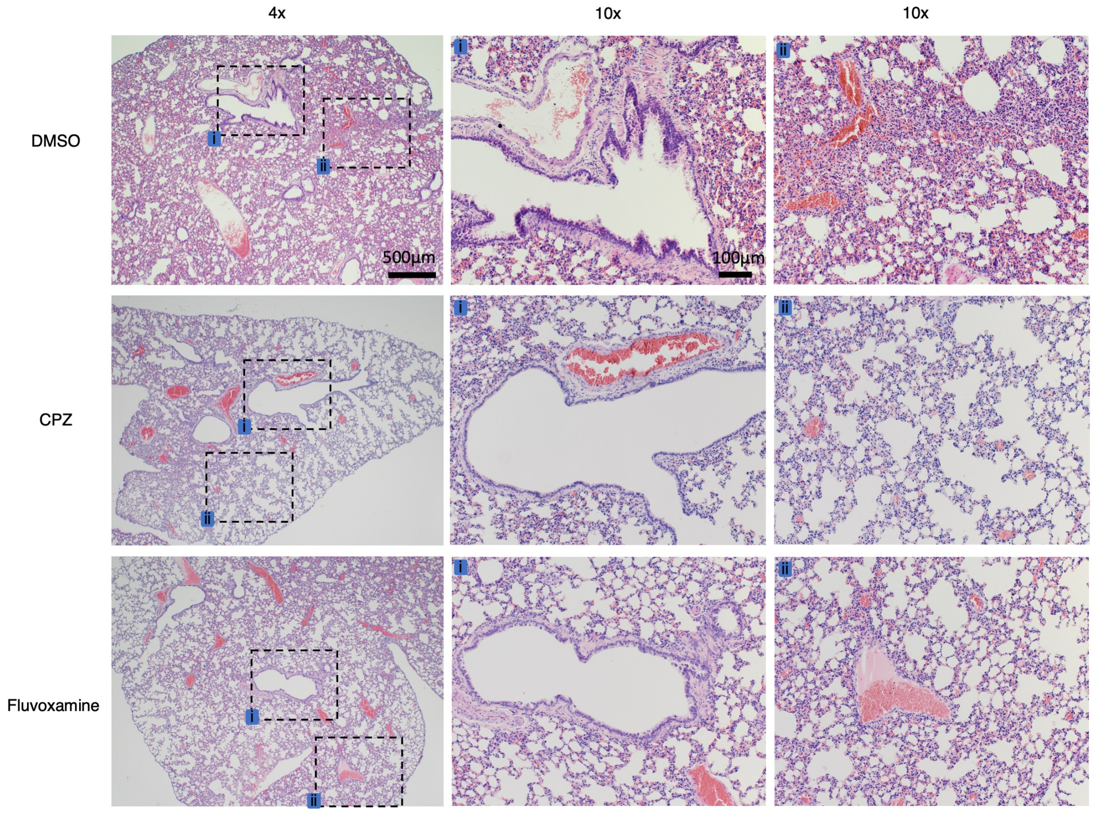

Supplementary Fig. 9 I Clathrin-mediated endocytosis inhibitors impede SARS-CoV-2 live virus-induced pathological damages in hamster lung tissues. Haematoxylin and eosin staining was used to examine the pathological damages of lung tissues after SARS-CoV-2 infection. The bronchiole epithelium region (i) and alveolar space region (ii) were highlighted in the images. In the DMSO-treated hamsters, extensive bronchiolar epithelium damages including bronchiolar epithelial desquamation and intense peribronchiolar mononuclear cell infiltration were detected. Alveolar congestion, haemorrhage and infiltration were observed in the alveolar region. CPZ and Fluvoxamine treatment alleviated the virus-induced pathological damages in regions of bronchiolar epithelium and alveolar space, including milder infiltration and alveolar haemorrhage.

Supplementary Table 1 l Constructs used in this study.

Constructs used in this study		
**construct ID**	**Design**	**Reference**
CHp178	pFUGW-CAGp-Spike(D614G)-GFP11-P2A-mCherry	this study
CHp229	pFUGW-CAGp-Spike(omicron)-GFP11-P2A-mCherry	this study
pBW93	pFUGW-CMVp-BSD-P2A-GFP1-10	this study
S_Q836V	pFUGW-CAGp-Spike(D614G, Q836V)-GFP11-P2A-mCherry	this study
S_Y837D	pFUGW-CAGp-Spike(D614G, Y837D)-GFP11-P2A-mCherry	this study
S_Y837F	pFUGW-CAGp-Spike(D614G, Y837F)-GFP11-P2A-mCherry	this study
S_Y837K	pFUGW-CAGp-Spike(D614G, Y837K)-GFP11-P2A-mCherry	this study
S_G838K	pFUGW-CAGp-Spike(D614G, G838K)-GFP11-P2A-mCherry	this study
S_G838R	pFUGW-CAGp-Spike(D614G, G838R)-GFP11-P2A-mCherry	this study
S_G838P	pFUGW-CAGp-Spike(D614G, G838P)-GFP11-P2A-mCherry	this study
S_D839Q	pFUGW-CAGp-Spike(D614G, D839Q)-GFP11-P2A-mCherry	this study
S_C840G	pFUGW-CAGp-Spike(D614G, C840G)-GFP11-P2A-mCherry	this study
S_L841H	pFUGW-CAGp-Spike(D614G, L841H)-GFP11-P2A-mCherry	this study
S_L841W	pFUGW-CAGp-Spike(D614G, L841W)-GFP11-P2A-mCherry	this study
S_D843G	pFUGW-CAGp-Spike(D614G, D843G)-GFP11-P2A-mCherry	this study
S_D843M	pFUGW-CAGp-Spike(D614G, D843M)-GFP11-P2A-mCherry	this study
S_D843Y	pFUGW-CAGp-Spike(D614G, D843Y)-GFP11-P2A-mCherry	this study
S_I844D	pFUGW-CAGp-Spike(D614G, I844D)-GFP11-P2A-mCherry	this study
S_I844M	pFUGW-CAGp-Spike(D614G, I844M)-GFP11-P2A-mCherry	this study
S_A845N	pFUGW-CAGp-Spike(D614G, A845N)-GFP11-P2A-mCherry	this study
S_A845T	pFUGW-CAGp-Spike(D614G, A845T)-GFP11-P2A-mCherry	this study
S_D843G	pFUGW-CAGp-Spike(D614G, D843G)-GFP11-P2A-mCherry	this study
S_D848H	pFUGW-CAGp-Spike(D614G, D848H)-GFP11-P2A-mCherry	this study
S_D848R	pFUGW-CAGp-Spike(D614G, D848R)-GFP11-P2A-mCherry	this study
S_D848T	pFUGW-CAGp-Spike(D614G, D848T)-GFP11-P2A-mCherry	this study
S_L849N	pFUGW-CAGp-Spike(D614G, L849N)-GFP11-P2A-mCherry	this study
S_I850H	pFUGW-CAGp-Spike(D614G, I850H)-GFP11-P2A-mCherry	this study
S_I850M	pFUGW-CAGp-Spike(D614G, I850M)-GFP11-P2A-mCherry	this study
S_A852M	pFUGW-CAGp-Spike(D614G, A852M)-GFP11-P2A-mCherry	this study
S_Q853R	pFUGW-CAGp-Spike(D614G, Q853R)-GFP11-P2A-mCherry	this study

S_Q853V	pFUGW-CAGp-Spike(D614G,Q853V)-GFP11-P2A-mCherry	this study
S_K854C	pFUGW-CAGp-Spike(D614G, K854C)-GFP11-P2A-mCherry	this study
S_K854L	pFUGW-CAGp-Spike(D614G, K854L)-GFP11-P2A-mCherry	this study
S_K854N	pFUGW-CAGp-Spike(D614G, K854N)-GFP11-P2A-mCherry	this study
S_K854Q	pFUGW-CAGp-Spike(D614G, K854Q)-GFP11-P2A-mCherry	this study
S_K854R	pFUGW-CAGp-Spike(D614G, K854R)-GFP11-P2A-mCherry	this study
S_K854T	pFUGW-CAGp-Spike(D614G, K854T)-GFP11-P2A-mCherry	this study
S_K854W	pFUGW-CAGp-Spike(D614G, K854W)-GFP11-P2A-mCherry	this study
S_K854H	pFUGW-CAGp-Spike(D614G, K854H)-GFP11-P2A-mCherry	this study
S_A846W	pFUGW-CAGp-Spike(D614G, A846W)-GFP11-P2A-mCherry	this study
S_C840S	pFUGW-CAGp-Spike(D614G, C840S)-GFP11-P2A-mCherry	this study
S_D848N	pFUGW-CAGp-Spike(D614G, D848N)-GFP11-P2A-mCherry	this study
S_C851S	pFUGW-CAGp-Spike(D614G, C851S)-GFP11-P2A-mCherry	this study
S_P681Y	pFUGW-CAGp-Spike(D614G, P681Y)-GFP11-P2A-mCherry	this study
S_R683H	pFUGW-CAGp-Spike(D614G, R683H)-GFP11-P2A-mCherry	this study
S_A684H	pFUGW-CAGp-Spike(D614G, A684H)-GFP11-P2A-mCherry	this study
S_R685F	pFUGW-CAGp-Spike(D614G, R685F)-GFP11-P2A-mCherry	this study
S_V687I	pFUGW-CAGp-Spike(D614G, V687I)-GFP11-P2A-mCherry	this study
S_A688T	pFUGW-CAGp-Spike(D614G, A688T)-GFP11-P2A-mCherry	this study
S_K1255F	pFUGW-CAGp-Spike(D614G, K1255F)-GFP11-P2A-mCherry	this study
O_K854H	pFUGW-CAGp-Spike(omicron, K854H)-GFP11-P2A-mCherry	this study
O_A846W	pFUGW-CAGp-Spike(omicron, A846W)-GFP11-P2A-mCherry	this study
O_C840S	pFUGW-CAGp-Spike(omicron, C840S)-GFP11-P2A-mCherry	this study
O_D848N	pFUGW-CAGp-Spike(omicron,D848N)-GFP11-P2A-mCherry	this study
O_C851S	pFUGW-CAGp-Spike(omicron, C851S)-GFP11-P2A-mCherry	this study
hFurin	pCMV3-huFurin	Sino biological (Cat No. HG10141-ACR)
hTMPRSS2	pCMV3-huTMPRSS2	Sino biological (Cat No. HG13070-CH)
AWp30	pFUGW-EFSp-Cas9-P2A-Zeo	Wong, A.S., et al., PNAS, 2016; 113(9):2544-9
pBW69d	pFUGW-hU6-ACE2_sg1-PGKp-puro-T2A-BFP	this study
pBW78d	pFUGW-hU6-safe harbor_sg1-PGKp-puro-T2A-BFP	this study
pBW111	pFUGW-hU6-ACE2_sg3-PGKp-puro-T2A-BFP	this study
pBW112	pFUGW-hU6-CAB39_sg1-PGKp-puro-T2A-BFP	this study
pBW113	pFUGW-hU6-CAB39_sg2-PGKp-puro-T2A-BFP	this study
pBW114	pFUGW-hU6-AP2M1_sg1-PGKp-puro-T2A-BFP	this study

pBW115	pFUGW-hU6-AP2M1_sg2-PGKp-puro-T2A-BFP	this study
pBW116	pFUGW-hU6-FCHO2_sg1-PGKp-puro-T2A-BFP	this study
pBW117	pFUGW-hU6-FCHO2_sg2-PGKp-puro-T2A-BFP	this study
pBW128	pFUGW-hU6-GBP6_sg1-PGKp-puro-T2A-BFP	this study
pBW129	pFUGW-hU6-GBP6_sg2-PGKp-puro-T2A-BFP	this study
pBW130	pFUGW-hU6-RNF2_sg1-PGKp-puro-T2A-BFP	this study
pBW131	pFUGW-hU6-RNF2_sg2-PGKp-puro-T2A-BFP	this study
pBW134	pFUGW-hU6-ZEB1_sg1-PGKp-puro-T2A-BFP	this study
pBW135	pFUGW-hU6-ZEB1_sg2-PGKp-puro-T2A-BFP	this study
pBW136	pFUGW-hU6-UBIAD1_sg1-PGKp-puro-T2A-BFP	this study
pBW137	pFUGW-hU6-UBIAD1_sg2-PGKp-puro-T2A-BFP	this study
pBW138	pFUGW-hU6-non targeting_sg1-PGKp-puro-T2A-BFP	this study
pBW139	pFUGW-hU6-non targeting_sg2-PGKp-puro-T2A-BFP	this study
pBW145	pFUGW-hU6-ACE2_sg-PGKp-puro-T2A-BFP (for vero E6 cells)	this study
pBW147	pFUGW-hU6-FCHO2_sg-PGKp-puro-T2A-BFP (for vero E6 cells)	this study
pBW168	pFUGW-hU6-NDUFB10_sg1-PGKp-puro-T2A-BFP	this study
pBW169	pFUGW-hU6-NDUFB10_sg2-PGKp-puro-T2A-BFP	this study
addgene 138152	pFUGW-EF1α-d2EGFP	Wessels, H.H., et al., Nat Biotechnol. 2020 Jun;38(6):722-727.
addgene 179907	pFUGW-CAGp-spike(omicron)	Garcia-Beltran, W.F., et al. Cell. 2022 Jan 6. pii: S0092-8674(21)01496-3.
addgene 10879	pLKO.1-TRC contorl	Moffat, J., et al.Cell, 2006. 124(6): p. 1283-98.
pBW164	pFUGW-hU6-CHC_sh1-PGKp-puro	this study
pBW165	pFUGW-hU6-CHC_sh2-PGKp-puro	this study
pBW166	pFUGW-hU6-CHC_sh3-PGKp-puro	this study
pBW167	pFUGW-hU6-non targeting_sh-PGKp-puro	this study
addgene 80409	pHR-SFFV-GFP1-10	Kamiyama, D. et al.Nat Commun 7, 11046 (2016).
pBW89	pFUGW-CMVp-BSD-P2A-GFP11x7	this study

Supplementary Table 2 | Primers used in this study.

List of primers and sgRNAs used		
**Primers**		
**Name**	**Sequence**	**Description**
BW275-F	ACACTCTTTCCCTACACGACGCTCTTCCGATCTCTTGTGGAAAGGACGAAACA	1st step PCR primers for amplication of the sgRNA library
BW276-R	GTGACTGGAGTTCAGACGTGTGCTCTTCCGATCTCTAAAGCGCATGCTCCAGAC	
Nova_i5_1	AATGATACGGCGACCACCGAGATCTACACCGATTGCAACACTCTTTCCCTACACGACGCTCTTCCGATC	2nd step PCR primers for addition of the illumina adapters and index sequence
Nova_i5_2	AATGATACGGCGACCACCGAGATCTACACGCAGAACAACACTCTTTCCCTACACGACGCTCTTCCGATC	
Nova_i5_3	AATGATACGGCGACCACCGAGATCTACACGCCTTCTTACACTCTTTCCCTACACGACGCTCTTCCGATC	
Nova_i5_4	AATGATACGGCGACCACCGAGATCTACACCGGAAGAAACACTCTTTCCCTACACGACGCTCTTCCGATC	
Nova_i5_5	AATGATACGGCGACCACCGAGATCTACACCCAGAGAAACACTCTTTCCCTACACGACGCTCTTCCGATC	
Nova_i5_6	AATGATACGGCGACCACCGAGATCTACACGAGGAGAAACACTCTTTCCCTACACGACGCTCTTCCGATC	
Nova_i5_7	AATGATACGGCGACCACCGAGATCTACACCACCAGAAACACTCTTTCCCTACACGACGCTCTTCCGATC	
Nova_i5_8	AATGATACGGCGACCACCGAGATCTACACACGAGGAAACACTCTTTCCCTACACGACGCTCTTCCGATC	
Nova_i5_9	AATGATACGGCGACCACCGAGATCTACACGGTAGGAAACACTCTTTCCCTACACGACGCTCTTCCGATC	
Nova_i5_10	AATGATACGGCGACCACCGAGATCTACACGAACGGAAACACTCTTTCCCTACACGACGCTCTTCCGATC	
Nova_i5_11	AATGATACGGCGACCACCGAGATCTACACTTGCGGAAACACTCTTTCCCTACACGACGCTCTTCCGATC	
Nova_i5_12	AATGATACGGCGACCACCGAGATCTACACCAGTGGAAACACTCTTTCCCTACACGACGCTCTTCCGATC	
Nova_i5_13	AATGATACGGCGACCACCGAGATCTACACTCCTGGAAACACTCTTTCCCTACACGACGCTCTTCCGATC	
Nova_i5_14	AATGATACGGCGACCACCGAGATCTACACTACGCGAAACACTCTTTCCCTACACGACGCTCTTCCGATC	
Nova_i5_15	AATGATACGGCGACCACCGAGATCTACACAGACCGAAACACTCTTTCCCTACACGACGCTCTTCCGATC	
Nova_i5_16	AATGATACGGCGACCACCGAGATCTACACTGGTCGAAACACTCTTTCCCTACACGACGCTCTTCCGATC	
Nova_i7_1	CAAGCAGAAGACGGCATACGAGATTTGGTGGAGTGACTGGAGTTCAGACGTGTGCTCTTCCGATC	
Nova_i7_2	CAAGCAGAAGACGGCATACGAGATTTGGCACTGTGACTGGAGTTCAGACGTGTGCTCTTCCGATC	
Nova_i7_3	CAAGCAGAAGACGGCATACGAGATTTGGATCCGTGACTGGAGTTCAGACGTGTGCTCTTCCGATC	
Nova_i7_4	CAAGCAGAAGACGGCATACGAGATTTGACGTCGTGACTGGAGTTCAGACGTGTGCTCTTCCGATC	
Nova_i7_5	CAAGCAGAAGACGGCATACGAGATTTGAGCTGGTGACTGGAGTTCAGACGTGTGCTCTTCCGATC	
Nova_i7_6	CAAGCAGAAGACGGCATACGAGATTTGAGGCAGTGACTGGAGTTCAGACGTGTGCTCTTCCGATC	
Nova_i7_7	CAAGCAGAAGACGGCATACGAGATTTATCGGCGTGACTGGAGTTCAGACGTGTGCTCTTCCGATC	
Nova_i7_8	CAAGCAGAAGACGGCATACGAGATTTACGGAGGTGACTGGAGTTCAGACGTGTGCTCTTCCGATC	
Nova_i7_9	CAAGCAGAAGACGGCATACGAGATTTACAGCCGTGACTGGAGTTCAGACGTGTGCTCTTCCGATC	
Nova_i7_10	CAAGCAGAAGACGGCATACGAGATTTAGCCGAGTGACTGGAGTTCAGACGTGTGCTCTTCCGATC	
Nova_i7_11	CAAGCAGAAGACGGCATACGAGATTCTCCAAGGTGACTGGAGTTCAGACGTGTGCTCTTCCGATC	
Nova_i7_12	CAAGCAGAAGACGGCATACGAGATTCTCGGATGTGACTGGAGTTCAGACGTGTGCTCTTCCGATC	
Nova_i7_13	CAAGCAGAAGACGGCATACGAGATTCTGTGCAGTGACTGGAGTTCAGACGTGTGCTCTTCCGATC	
Nova_i7_14	CAAGCAGAAGACGGCATACGAGATTCTGCCATGTGACTGGAGTTCAGACGTGTGCTCTTCCGATC	
Nova_i7_15	CAAGCAGAAGACGGCATACGAGATTCTGGAACGTGACTGGAGTTCAGACGTGTGCTCTTCCGATC	
Nova_i7_16	CAAGCAGAAGACGGCATACGAGATTCTGAGGTGTGACTGGAGTTCAGACGTGTGCTCTTCCGATC	
SA160	AATGATACGGCGACCACCGA	

SA161	CAAGCAGAAGACGGCATACGA	quantification of NGS library concentration
S_DMSfs1	TTACCTCGTCTCTTGCTTCTTACCAGACCCAAACCAACTCTCCTAGGCGAGCCCGGTCCGTAGCCA	universal foward primer for FPPR DMS library construction
S_DMSrs	TTACCTCGTCTCTTAAATTTTTGGGCGCATATCAGATCC	universal reverse primer for Furin cleavage site DMS library construction
S_DMS1	TTCGTCTCTTGCTNNSTACCAGACCCAAACCAACTCTCCTAGGCGTGCCCGGTCCGTAGCCAGTCAAAGCATAATTGCGTACACCATGAG	Furin cleavage site_NNS primer
S_DMS2	TTCGTCTCTTGCTTCTNNSCAGACCCAAACCAACTCTCCTAGGCGTGCCCGGTCCGTAGCCAGTCAAAGCATAATTGCGTACACCATGAG	
S_DMS3	TTCGTCTCTTGCTTCTTACNNSACCCAAACCAACTCTCCTAGGCGTGCCCGGTCCGTAGCCAGTCAAAGCATAATTGCGTACACCATGAG	
S_DMS4	TTCGTCTCTTGCTTCTTACCAGNNSCAAACCAACTCTCCTAGGCGTGCCCGGTCCGTAGCCAGTCAAAGCATAATTGCGTACACCATGAG	
S_DMS5	TTCGTCTCTTGCTTCTTACCAGACCNNSACCAACTCTCCTAGGCGTGCCCGGTCCGTAGCCAGTCAAAGCATAATTGCGTACACCATGAG	
S_DMS6	TTCGTCTCTTGCTTCTTACCAGACCCAANNSAACTCTCCTAGGCGTGCCCGGTCCGTAGCCAGTCAAAGCATAATTGCGTACACCATGAG	
S_DMS7	TTCGTCTCTTGCTTCTTACCAGACCCAAACCNNSTCTCCTAGGCGTGCCCGGTCCGTAGCCAGTCAAAGCATAATTGCGTACACCATGAG	
S_DMS8	TTCGTCTCTTGCTTCTTACCAGACCCAAACCAACNNSCCTAGGCGTGCCCGGTCCGTAGCCAGTCAAAGCATAATTGCGTACACCATGAG	
S_DMS9	TTCGTCTCTTGCTTCTTACCAGACCCAAACCAACTCTNNSAGGCGTGCCCGGTCCGTAGCCAGTCAAAGCATAATTGCGTACACCATGAG	
S_DMS10	TTCGTCTCTTGCTTCTTACCAGACCCAAACCAACTCTCCTNNSCGTGCCCGGTCCGTAGCCAGTCAAAGCATAATTGCGTACACCATGAG	
S_DMS11	TTCGTCTCTTGCTTCTTACCAGACCCAAACCAACTCTCCTAGGNNSGCCCGGTCCGTAGCCAGTCAAAGCATAATTGCGTACACCATGAG	
S_DMS12	TTCGTCTCTTGCTTCTTACCAGACCCAAACCAACTCTCCTAGGCGTNNSCGGTCCGTAGCCAGTCAAAGCATAATTGCGTACACCATGAG	
S_DMS13	TTCGTCTCTTGCTTCTTACCAGACCCAAACCAACTCTCCTAGGCGTGCCNNSTCCGTAGCCAGTCAAAGCATAATTGCGTACACCATGAG	
S_DMS14	TTCGTCTCTTGCTTCTTACCAGACCCAAACCAACTCTCCTAGGCGTGCCCGGNNSGTAGCCAGTCAAAGCATAATTGCGTACACCATGAG	
S_DMS15	TTCGTCTCTTGCTTCTTACCAGACCCAAACCAACTCTCCTAGGCGTGCCCGGTCCNNSGCCAGTCAAAGCATAATTGCGTACACCATGAG	
S_DMS16	TTCGTCTCTTGCTTCTTACCAGACCCAAACCAACTCTCCTAGGCGTGCCCGGTCCGTANNSAGTCAAAGCATAATTGCGTACACCATGAG	
S_DMS17	TTCGTCTCTTGCTTCTTACCAGACCCAAACCAACTCTCCTAGGCGTGCCCGGTCCGTAGCCNNSCAAAGCATAATTGCGTACACCATGAG	
S_DMS18	TTCGTCTCTTGCTTCTTACCAGACCCAAACCAACTCTCCTAGGCGTGCCCGGTCCGTAGCCAGTNNSAGCATAATTGCGTACACCATGAG	
S_DMS19	TTCGTCTCTTGCTTCTTACCAGACCCAAACCAACTCTCCTAGGCGTGCCCGGTCCGTAGCCAGTCAANNSATAATTGCGTACACCATGAG	
S_DMS20	TTCGTCTCTTAAASNNTTGGGCGCATATCAGATCCCGCGCCGCGATGTCGCCCAAACAATCACCGTATTGTTTAATAAAGCCAGCATCGG	FPPR_NNS primer
S_DMS21	TTCGTCTCTTAAATTTSNNGGCGCATATCAGATCCCGCGCCGCGATGTCGCCCAAACAATCACCGTATTGTTTAATAAAGCCAGCATCGG	
S_DMS22	TTCGTCTCTTAAATTTTTGSNNGCATATCAGATCCCGCGCCGCGATGTCGCCCAAACAATCACCGTATTGTTTAATAAAGCCAGCATCGG	
S_DMS23	TTCGTCTCTTAAATTTTTGGGCSNNTATCAGATCCCGCGCCGCGATGTCGCCCAAACAATCACCGTATTGTTTAATAAAGCCAGCATCGG	
S_DMS24	TTCGTCTCTTAAATTTTTGGGCGCASNNCAGATCCCGCGCCGCGATGTCGCCCAAACAATCACCGTATTGTTTAATAAAGCCAGCATCGG	
S_DMS25	TTCGTCTCTTAAATTTTTGGGCGCATATSNNATCCCGCGCCGCGATGTCGCCCAAACAATCACCGTATTGTTTAATAAAGCCAGCATCGG	
S_DMS26	TTCGTCTCTTAAATTTTTGGGCGCATATCAGSNNCCGCGCCGCGATGTCGCCCAAACAATCACCGTATTGTTTAATAAAGCCAGCATCGG	
S_DMS27	TTCGTCTCTTAAATTTTTGGGCGCATATCAGATCSNNCGCCGCGATGTCGCCCAAACAATCACCGTATTGTTTAATAAAGCCAGCATCGG	
S_DMS28	TTCGTCTCTTAAATTTTTGGGCGCATATCAGATCCCGSNNCGCGATGTCGCCCAAACAATCACCGTATTGTTTAATAAAGCCAGCATCGG	
S_DMS29	TTCGTCTCTTAAATTTTTGGGCGCATATCAGATCCCGCGCSNNGATGTCGCCCAAACAATCACCGTATTGTTTAATAAAGCCAGCATCGG	
S_DMS30	TTCGTCTCTTAAATTTTTGGGCGCATATCAGATCCCGCGCCGCSNNGTCGCCCAAACAATCACCGTATTGTTTAATAAAGCCAGCATCGG	
S_DMS31	TTCGTCTCTTAAATTTTTGGGCGCATATCAGATCCCGCGCCGCGATSNNGCCCAAACAATCACCGTATTGTTTAATAAAGCCAGCATCGG	
S_DMS32	TTCGTCTCTTAAATTTTTGGGCGCATATCAGATCCCGCGCCGCGATGTCSNNCAAACAATCACCGTATTGTTTAATAAAGCCAGCATCGG	
S_DMS33	TTCGTCTCTTAAATTTTTGGGCGCATATCAGATCCCGCGCCGCGATGTCGCCSNNACAATCACCGTATTGTTTAATAAAGCCAGCATCGG	
S_DMS34	TTCGTCTCTTAAATTTTTGGGCGCATATCAGATCCCGCGCCGCGATGTCGCCCAASNNATCACCGTATTGTTTAATAAAGCCAGCATCGG	

S_DMS35	TTCGTCTCTTAAATTTTTGGGCGCATATCAGATCCCGCGCCGCGATGTCGCCCAAACASN<br>NACCGTATTGTTTAATAAAGCCAGCATCGG	
S_DMS36	TTCGTCTCTTAAATTTTTGGGCGCATATCAGATCCCGCGCCGCGATGTCGCCCAAACAAT<br>CSNNGTATTGTTTAATAAAGCCAGCATCGG	
S_DMS37	TTCGTCTCTTAAATTTTTGGGCGCATATCAGATCCCGCGCCGCGATGTCGCCCAAACAAT<br>CACCSNNTTGTTTAATAAAGCCAGCATCGG	
S_DMS38	TTCGTCTCTTAAATTTTTGGGCGCATATCAGATCCCGCGCCGCGATGTCGCCCAAACAAT<br>CACCGTASNNTTTAATAAAGCCAGCATCGG	
C508f	CACGACGCTCTTCCGATCTCATGTGAACAATTCATACGAATGTG	1st step PCR primers for amplication of the Furin DMS library
C509r	CAGACGTGTGCTCTTCCGATCTATTTGTTGGGATGGCAATGGAG	
C512f	CACGACGCTCTTCCGATCTCGGACCCCAGTAAACCCTC	1st step PCR primers for amplication of the FPPR DMS library
C513r	CAGACGTGTGCTCTTCCGATCTCCGAATGTCCATCCAGACGTT	
S_K854H	TTCGTCTCTTAAAGTGTTGGGCGCATATCAGATCCCGCGCCGCGATGTCGCCCAAACAAT<br>CACCGTATTGTTTAATAAAGCCAGCATCGG	
S_A846W	TTCGTCTCTTAAATTTTTGGGCGCATATCAGATCCCGCCACGCGATGTCGCCCAAACAAT<br>CACCGTATTGTTTAATAAAGCCAGCATCGG	
S_K854C	TTCGTCTCTTAAAGCATTGGGCGCATATCAGATCCCGCGCCGCGATGTCGCCCAAACAAT<br>CACCGTATTGTTTAATAAAGCCAGCATCGG	
S_A852M	TTCGTCTCTTAAATTTTTGCATGCATATCAGATCCCGCGCCGCGATGTCGCCCAAACAAT<br>CACCGTATTGTTTAATAAAGCCAGCATCGG	
S_K854L	TTCGTCTCTTAAACAGTTGGGCGCATATCAGATCCCGCGCCGCGATGTCGCCCAAACAAT<br>CACCGTATTGTTTAATAAAGCCAGCATCGG	
S_Q853V	TTCGTCTCTTAAATTTCACGGCGCATATCAGATCCCGCGCCGCGATGTCGCCCAAACAAT<br>CACCGTATTGTTTAATAAAGCCAGCATCGG	
S_K854N	TTCGTCTCTTAAAGTTTTGGGCGCATATCAGATCCCGCGCCGCGATGTCGCCCAAACAAT<br>CACCGTATTGTTTAATAAAGCCAGCATCGG	
S_D843M	TTCGTCTCTTAAATTTTTGGGCGCATATCAGATCCCGCGCCGCGATCATGCCCAAACAAT<br>CACCGTATTGTTTAATAAAGCCAGCATCGG	
S_G838Y	TTCGTCTCTTAAATTTTTGGGCGCATATCAGATCCCGCGCCGCGATGTCGCCCAAACAAT<br>CGTAGTATTGTTTAATAAAGCCAGCATCGG	
S_Y837F	TTCGTCTCTTAAATTTTTGGGCGCATATCAGATCCCGCGCCGCGATGTCGCCCAAACAAT<br>CACCGAATTGTTTAATAAAGCCAGCATCGG	
S_K854W	TTCGTCTCTTAAACCATTGGGCGCATATCAGATCCCGCGCCGCGATGTCGCCCAAACAAT<br>CACCGTATTGTTTAATAAAGCCAGCATCGG	
S_C840S	TTCGTCTCTTAAATTTTTGGGCGCATATCAGATCCCGCGCCGCGATGTCGCCCAAGCTAT<br>CACCGTATTGTTTAATAAAGCCAGCATCGG	
S_D848N	TTCGTCTCTTAAATTTTTGGGCGCATATCAGGTTCCGCGCCGCGATGTCGCCCAAACAAT<br>CACCGTATTGTTTAATAAAGCCAGCATCGG	
S_C851S	TTCGTCTCTTAAATTTTTGGGCGCTTATCAGATCCCGCGCCGCGATGTCGCCCAAACAAT<br>CACCGTATTGTTTAATAAAGCCAGCATCGG	
S_P681Y	TTCGTCTCTTGCTTCTTACCAGACCCAAACCAACTCTTACAGGCGTGCCCGGTCCGTAGC<br>CAGTCAAAGCATAATTGCGTACACCATGAG	
S_R683H	TTCGTCTCTTGCTTCTTACCAGACCCAAACCAACTCTCCTAGGCACGCCCGGTCCGTAGC<br>CAGTCAAAGCATAATTGCGTACACCATGAG	
S_A684H	TTCGTCTCTTGCTTCTTACCAGACCCAAACCAACTCTCCTAGGCGACACCGGTCCGTAGC<br>CAGTCAAAGCATAATTGCGTACACCATGAG	
S_R685F	TTCGTCTCTTGCTTCTTACCAGACCCAAACCAACTCTCCTAGGCGTGCCTTCTCCGTAGC<br>CAGTCAAAGCATAATTGCGTACACCATGAG	
S_V687I	TTCGTCTCTTGCTTCTTACCAGACCCAAACCAACTCTCCTAGGCGTGCCCGGTCCATCGC<br>CAGTCAAAGCATAATTGCGTACACCATGAG	
S_A688T	TTCGTCTCTTGCTTCTTACCAGACCCAAACCAACTCTCCTAGGCGTGCCCGGTCCGTAAC<br>CAGTCAAAGCATAATTGCGTACACCATGAG	
O_K854H	TTCGTCTCTTGAAGTGTTGAGCACAGATAAGGTCGC	
O_A846W	TTCGTCTCTTGAATTTTTGAGCACAGATAAGGTCGCGCCACGCAATGTCTCCCAAACAAT<br>C	
O_C840S	TTCGTCTCTTGAATTTTTGAGCACAGATAAGGTCGCGGGCCGCAATGTCTCCCAAGCTAT<br>CCCCATACTGCTTAATGAAAC	
O_D848N	TTCGTCTCTTGAATTTTTGAGCACAGATAAGGTTGCGGGCCGCAATGTCT	
O_C851S	TTCGTCTCTTGAATTTTTGAGCGCTGATAAGGTCGCGGGCCG	
BW588	GTCTTTCTCGCTACCTGGTACG	qRT-PCR primer for CHC, RdRp, GAPDH(human), and β-actin (hamster)
BW589	GGTCCTGAGTCTCAGACAAAGC	
RdRp-F	CGCATACAGTCTTRCAGGCT	
RdRp-R	GTGTGATGTTGAWATGACATGGTC	
probe	FAM-TTAAGATGTGGTGCTTGCATACGTAGAC-IABkFQ	
GAPDH-F	ATTCCACCCATGGCAAATTC	
GAPDH-R	CGCTCCTGGAAGATGGTGAT	
β-actin-<br>F(hamster)	ACGGCCAGGTCATCACTATTG	

β-actin-R(hamster)	CAAGAAGGAAGGCTGGAAAAG	
