# Supplementary material for: High-throughput screening of genetic and cellular drivers of syncytium formation induced by the spike protein of SARS-CoV-2
Source: Nat Biomed Eng. 2023 Nov 23;8(3):291–309. doi: 10.1038/s41551-023-01140-z (PMC10963270; doi:10.1038/s41551-023-01140-z)
Supplement: Supplementary file 2 — Reporting Summary [file 41551_2023_1140_MOESM2_ESM.pdf]

nature portfolio

Corresponding author(s): Alan S. L. Wong

Last updated by author(s): Sep 8, 2023

# Reporting Summary

Nature Portfolio wishes to improve the reproducibility of the work that we publish. This form provides structure for consistency and transparency in reporting. For further information on Nature Portfolio policies, see our Editorial Policies and the Editorial Policy Checklist.

## Statistics

For all statistical analyses, confirm that the following items are present in the figure legend, table legend, main text, or Methods section.

| n/a | Confirmed | |
|---|---|---|
| ☐ | ☒ | The exact sample size (*n*) for each experimental group/condition, given as a discrete number and unit of measurement |
| ☐ | ☒ | A statement on whether measurements were taken from distinct samples or whether the same sample was measured repeatedly |
| ☐ | ☒ | The statistical test(s) used AND whether they are one- or two-sided<br>*Only common tests should be described solely by name; describe more complex techniques in the Methods section.* |
| ☒ | ☐ | A description of all covariates tested |
| ☒ | ☐ | A description of any assumptions or corrections, such as tests of normality and adjustment for multiple comparisons |
| ☐ | ☒ | A full description of the statistical parameters including central tendency (e.g. means) or other basic estimates (e.g. regression coefficient) AND variation (e.g. standard deviation) or associated estimates of uncertainty (e.g. confidence intervals) |
| ☐ | ☒ | For null hypothesis testing, the test statistic (e.g. *F*, *t*, *r*) with confidence intervals, effect sizes, degrees of freedom and *P* value noted<br>*Give P values as exact values whenever suitable.* |
| ☒ | ☐ | For Bayesian analysis, information on the choice of priors and Markov chain Monte Carlo settings |
| ☒ | ☐ | For hierarchical and complex designs, identification of the appropriate level for tests and full reporting of outcomes |
| ☐ | ☒ | Estimates of effect sizes (e.g. Cohen's *d*, Pearson's *r*), indicating how they were calculated |

*Our web collection on statistics for biologists contains articles on many of the points above.*

## Software and code

Policy information about availability of computer code

| | |
|---|---|
| Data collection | High-throughput sequencing data were collected using Illumina NovaSeq 6000 with NovaSeq Control software. Flow-cytometry data were collected using the BD FACSDiva(v9.0) software. High-throughput imaging data were collected using GE IN Cell Analyzer 6500HS. |
| Data analysis | DiMSum was used for analysing the Spike DMS NGS data (https://github.com/lehner-lab/DiMSum). MAGeCK (https://sourceforge.net/p/mageck/wiki/Home/) and JACKS (https://github.com/felicityallen/JACKS) were used to analyse the genome-wide CRISPR screen data. Kallisto (https://github.com/pachterlab/kallisto), tximport (https://github.com/mikelove/tximport), and DESeq2(http://bioconductor.org/packages/devel/bioc/vignettes/DESeq2/inst/doc/DESeq2.html#input-data) were used to analyse the RNA-seq data. Flowjo (version 10.8.1) was used to analyse data generated from the flow-cytometry experiments. Prism (v9) was used to plot the bar plots and to perform one-way ANOVA tests. R (v2021.09.2+382) with packages ggplot2 (v3.3.6) and tidyverse (v1.3.2) was used for plotting the scatter plots and heatmaps. R (v2021.09.2+382) with packages clusterProfiler(v.4.4.4) was used for GO analysis. GE IN Carta image analysing software(v2.x) and ImageJ (v1.53u) was used for the quantification of total area of green fluorescence. PyMOL (v2.5.2) was used for the molecular modelling and visualization of protein models. |

For manuscripts utilizing custom algorithms or software that are central to the research but not yet described in published literature, software must be made available to editors and reviewers. We strongly encourage code deposition in a community repository (e.g. GitHub). See the Nature Portfolio guidelines for submitting code & software for further information.

## Data

Policy information about availability of data

All manuscripts must include a data availability statement. This statement should provide the following information, where applicable:

- Accession codes, unique identifiers, or web links for publicly available datasets
- A description of any restrictions on data availability
- For clinical datasets or third party data, please ensure that the statement adheres to our policy

The main data supporting the results in this study are available within the paper and its Supplementary Information. The molecular structures of the Spike proteins of the SARS-CoV-2 variants are available from the Protein Data Bank, with accession codes 6XR8, 7KRQ and 7TO4. Source data for the figures are provided with this paper. The raw and analysed datasets generated during the study are available for research purposes from the corresponding authors on reasonable request.

## Human research participants

Policy information about studies involving human research participants and Sex and Gender in Research.

| | |
|---|---|
| Reporting on sex and gender | The study did not involve human research participants. |
| Population characteristics | — |
| Recruitment | — |
| Ethics oversight | — |

Note that full information on the approval of the study protocol must also be provided in the manuscript.

# Field-specific reporting

Please select the one below that is the best fit for your research. If you are not sure, read the appropriate sections before making your selection.

☒ Life sciences ☐ Behavioural & social sciences ☐ Ecological, evolutionary & environmental sciences

For a reference copy of the document with all sections, see nature.com/documents/nr-reporting-summary-flat.pdf

# Life sciences study design

All studies must disclose on these points even when the disclosure is negative.

| | |
|---|---|
| Sample size | Sample sizes were chosen to be able to show reproducibility and statistical significance. No methods were used to predetermine sample size. >500-fold more cells for lentiviral infection than the size of the library being tested in genetic screens were used to ensure high fold-representation. From the NGS data, >98% coverage of the variants could be achieved with this sample size. |
| Data exclusions | It was previously reported that filtering of the genetic screen data to remove library members with low representation in the reference set resulted in a reduced false-negative rate (Sim et al., Genome Biol. 2011; 12(10): R104). Yet, the exclusion criteria has not been standardized and thus were not pre-established. For Illumina sequencing data from the Spike DMS screens and the genome-wide CRISPR screen in this study, only single variants or sgRNAs that gave more than 50 absolute reads in the unsorted population were analysed to improve data reliability. |
| Replication | All data were reliably reproduced. The methods and materials used in our experiments are described to facilitate replication.Transfection of of the onstructs into human cells was performed independently to produce biological replicates. Infected cell pools were sorted into bins independently to produce biological replicates for genomic-DNA extraction for NGS sequencing. All biological replicates were analysed independently, and replicate numbers are provided in the text and figure legends. |
| Randomization | No randomization was used for samples, as samples with particular genetic constituents were needed for the experiments. During the construction of mutant libraries, cell culture, transfection, infection, cell sorting, sample preparation for NGS sequencing and data analysis, samples were not grouped in a way relating to the identity of the sample. The timing of when samples were ready determined the grouping of the samples in sequencing runs. |
| Blinding | Blinding was not relevant to the study, as samples with particular genetic constituents were needed for the experiments. Sample labelling was used to prevent mixing up experimental samples. |

# Reporting for specific materials, systems and methods

We require information from authors about some types of materials, experimental systems and methods used in many studies. Here, indicate whether each material, system or method listed is relevant to your study. If you are not sure if a list item applies to your research, read the appropriate section before selecting a response.

## Materials & experimental systems

| n/a | Involved in the study | |
|---|---|---|
| ☐ | ☒ | Antibodies |
| ☐ | ☒ | Eukaryotic cell lines |
| ☒ | ☐ | Palaeontology and archaeology |
| ☐ | ☒ | Animals and other organisms |
| ☒ | ☐ | Clinical data |
| ☒ | ☐ | Dual use research of concern |

## Methods

| n/a | Involved in the study | |
|---|---|---|
| ☒ | ☐ | ChIP-seq |
| ☐ | ☒ | Flow cytometry |
| ☒ | ☐ | MRI-based neuroimaging |

# Antibodies

Antibodies used

Rabbit Anti-SARS-CoV-2 Spike (944-1214aa) polyclonal antibodies: https://www.ptglab.com/products/spike-protein-944-1214aa-Antibody-28867-1-AP.htm
Supplier name: Proteintech
Catalog number: 28867-1-AP
Dilution: 1:500

SARS-CoV-2 Spike S1 Subunit Alexa Fluor® 488-conjugated Antibody
https://www.rndsystems.com/products/sars-cov-2-spike-s1-subunit-alexa-fluor-488-conjugated-antibody-1035206_fab105403g
Supplier name: R&D
Clone #1035206
Catalog number: FAB105403G
Dilution: 1:200

Goat Anti-ACE2 polyclonal antibodies: https://www.rndsystems.com/products/human-mouse-rat-hamster-ace-2-antibody_af933
Supplier name: R&D Systems
Catalog number: AF933
Dilution: 1:100

Donkey anti-Goat IgG(H+L) Cross-Adsorbed Secondary Antibody-AF568: https://www.thermofisher.com/antibody/product/Donkey-anti-Goat-IgG-H-L-Cross-Adsorbed-Secondary-Antibody-Polyclonal/A-11057
Supplier name: Thermo Fisher
Catalog number: A-11057
Dilution: 1:1000

Goat anti-Rabbit IgG(H+L) Cross-Adsorbed Secondary Antibody-AF488: https://www.thermofisher.com/antibody/product/Goat-anti-Rabbit-IgG-H-L-Cross-Adsorbed-Secondary-Antibody-Polyclonal/A-11008
Supplier name: Thermo Fisher
Catalog number: A-11008
Dilution: 1:1000

in-house rabbit anti-SARS-CoV-2 N antibody

in-house guinea pig anti-SARS-Cov-2 N antibody

Recombinant Anti-Sodium Potassium ATPase antibody https://www.abcam.com/products/primary-antibodies/sodium-potassium-atpase-antibody-ep1845y-plasma-membrane-loading-control-ab76020.html
Supplier name: abcam
Catalog number: ab76020
Dilution: 1:500

Goat anti-Guinea Pig IgG (H+L) Highly Cross-Adsorbed Secondary Antibody, Alexa Fluor™ 488
https://www.thermofisher.com/antibody/product/Goat-anti-Guinea-Pig-IgG-H-L-Highly-Cross-Adsorbed-Secondary-Antibody-Polyclonal/A-11073
Supplier name: Thermo Fisher
Catalog number: A-11073
Dilution: 1:1000

Goat anti-Rabbit IgG (H+L) Cross-Adsorbed Secondary Antibody, Alexa Fluor™ 568
https://www.thermofisher.com/antibody/product/Goat-anti-Rabbit-IgG-H-L-Cross-Adsorbed-Secondary-Antibody-Polyclonal/A-11011
Supplier name: Thermo Fisher
Catalog number: A-11011
Dilution: 1:1000

Anti-Clathrin heavy chain antibody (ab21679)
https://www.abcam.com/products/primary-antibodies/clathrin-heavy-chain-antibody-ab21679.html

Supplier name: abcam
Catalog number: ab21679
Dilution: 1:900

GAPDH (14C10) Rabbit mAb #2118
https://www.cellsignal.com/products/primary-antibodies/gapdh-14c10-rabbit-mab/2118
Supplier name: Cell Signaling
Catalog number: 2118
Dilution: 1:5000

Anti-rabbit IgG, HRP-linked Antibody #7074
https://www.cellsignal.com/products/secondary-antibodies/anti-rabbit-igg-hrp-linked-antibody/7074
Supplier name: Cell Signaling
Catalog number: 7074
Dilution: 1:10000

Validation

Polyclonal anti-SARS-CoV-2 Spike antibodies recognize an epitope located on the S2 (944-1214aa) of the Spike protien. The antibodies are validated by the commercial vendor. (https://www.ptglab.com/products/pictures/pdf/28867-1-AP.pdf)

Monoclonal anti-SARS-Cov-2 spike S1 antibody is validated by the commercial vendor. (https://resources.rndsystems.com/pdfs/datasheets/fab105403g.pdf?v=20230516&_ga=2.4377843.1359546541.1684248237-1703500799.1684248237&_gac=1.82504036.1684248259.CjwKCAjw04yjBhApEiwAJcvNoSpk9L07LH1ShqLhoNpqEDdgtriMW5aU7QzupvzVfEWLos7SFuWu-hoCADkQAvD_BwE)

Polyclonal anti-ACE2 antibodies recognize human/mouse/rat/hamster ACE2. The antibodies are validated for flow cytometry, WB, and IHC by the commercial vendor. (https://resources.rndsystems.com/pdfs/datasheets/af933.pdf?v=20221204&_ga=2.166088636.324426401.1670231890-376477508.1670231890&_gac=1.242411958.1670231890.EAIaIQobChMI_KaG0ZLi-wIVRNeWCh0nVgB2EAAYASAAEgInj_D_BwE)

The in-house rabbit anti-SARS-CoV-2 N antibody and guinea pig anti-SARS-Cov-2 N antibody were validated immunofluorescence staining in the previous publication (PMID: 36662861)

Recombinant Anti-Sodium Potassium ATPase antibody recognizes an intracellular epitope of Sodium/potassium-transporting ATPase alpha-1 subunit. This antibody is recommended for ICC, Flow Cytometry, WB, and IHC. This antibody is validated by the commercial vendor. (file:///C:/Users/bwang/Downloads/datasheet_76020.pdf)

Anti-Clathrin heavy chain antibody is recommended for WB, IHC, and ICC. This antibody is validated by the commercial vendor. (file:///C:/Users/bwang/Downloads/datasheet_21679.pdf)

GAPDH (14C10) Rabbit mAb is used to detect endogenous levels of total GAPDH protein in Human, Mouse, Rat, Monkey, Bovine, Pig. It is recommended for WB, IHC, IF, and Flow Cytometry. This antibody is validated by the commercial vendor. (https://www.cellsignal.com/products/primary-antibodies/gapdh-14c10-rabbit-mab/2118)

Anti-rabbit IgG, HRP-linked Antibody is used for chemiluminescent detection. This antibody is validated by the commercial vendor. (https://www.cellsignal.com/products/secondary-antibodies/anti-rabbit-igg-hrp-linked-antibody/7074)

Goat anti-Guinea Pig IgG(H+L) Secondary Antibody-AF488 is recommended for ICC/IF and IHC. This antibody is validated by the commercial vendor. (https://www.thermofisher.com/order/genome-database/dataSheetPdf?producttype=antibody&productsubtype=antibody_secondary&productId=A-11073&version=300)

Goat anti-Rabbit IgG(H+L) Secondary Antibody-AF568 is recommended for Flow Cytometry, ICC/IF and IHC. This antibody is validated by the commercial vendor. (https://www.thermofisher.com/order/genome-database/dataSheetPdf?producttype=antibody&productsubtype=antibody_secondary&productId=A-11011&version=300)

anti-Goat IgG(H+L) Secondary Antibody-AF568 is recommended for flow cytometry, IHC, and ICC/IF. This antibody is validated by the commercial vendor. (https://www.thermofisher.com/order/genome-database/dataSheetPdf?producttype=antibody&productsubtype=antibody_secondary&productId=A-11057&version=271)

anti-rabbit IgG(H+L) Secondary Antibody-AF488 is recommended for flow cytometry, IHC, and ICC/IF. This antibody is validated by the commercial vendor. (https://www.thermofisher.com/order/genome-database/dataSheetPdf?producttype=antibody&productsubtype=antibody_secondary&productId=A-11008&version=271)

## Eukaryotic cell lines

Policy information about cell lines and Sex and Gender in Research

Cell line source(s)

HEK293T and Vero E6 cells were obtained from American Type Culture Collection (ATCC). VeroE6-TMPRSS2 was obtained from the Japanese Collection of Research Bioresources Cell Bank. A549-ACE2 cells were obtained from InvivoGen https://www.invivogen.com/a549-ace2-tmprss2-cells.

Authentication

HEK293T cells were authenticated by STR profiling by the commercial vendor. A549-ACE2, Vero E6 and Vero E6-TMPRSS2 cell lines were not authenticated after receiving them.

Mycoplasma contamination

Mycoplasma contamination was tested and confirmed to be negative. All cell-culture medium was supplemented with antibiotic-antimycotic solution to prevent bacterial and fungal contamination.

| | |
|---|---|
| Commonly misidentified lines (See ICLAC register) | No commonly misidentified cell lines were used (HEK293T, A549 and Vero E6 cells are not included in the ICLAC register). |

## Animals and other research organisms

Policy information about studies involving animals; ARRIVE guidelines recommended for reporting animal research, and Sex and Gender in Research

| | |
|---|---|
| Laboratory animals | Syrian hamsters (4 to 6 weeks old, male) were obtained from the Chinese University of Hong Kong Laboratory Animal Service Centre through the HKU Centre for Comparative Medicine Research. |
| Wild animals | The study did not involve wild animals. |
| Reporting on sex | Male Golden Syrian hamsters. |
| Field-collected samples | The study did not involve samples collected from the field. |
| Ethics oversight | The animal studies were approved by the Committee on the Use of Live Animals in Teaching and Research of The University of Hong Kong. |

Note that full information on the approval of the study protocol must also be provided in the manuscript.

## Flow Cytometry

### Plots

Confirm that:

☒ The axis labels state the marker and fluorochrome used (e.g. CD4-FITC).

☒ The axis scales are clearly visible. Include numbers along axes only for bottom left plot of group (a 'group' is an analysis of identical markers).

☒ All plots are contour plots with outliers or pseudocolor plots.

☒ A numerical value for number of cells or percentage (with statistics) is provided.

### Methodology

| | |
|---|---|
| Sample preparation | Cell cultures were treated with trypsin and diluted in complete media or PBS for flow-cytometry experiments. |
| Instrument | BD LSRFortessaTM, ACEA NovoCyte Quanteon, Agilent NovoCyte Advanteon BVYG were used for data collection. Cell sorting was performed on a BD Influx cell sorter. |
| Software | All cytometry data were analysed by FlowJo (v10.8.1). |
| Cell population abundance | Drop delay was determined using BD Accudrop beads. Cells were filtered through nylon mesh filters before sorting through a 100-µm nozzle using 1.0 Drop Pure sorting mode. Details are described in Methods. |
| Gating strategy | Viable and intact cells were gated from FSC/SSC for analysis. Details are described in Methods. |

☒ Tick this box to confirm that a figure exemplifying the gating strategy is provided in the Supplementary Information.
