# Supplementary figures and images for: High-throughput screening of genetic and cellular drivers of syncytium formation induced by the spike protein of SARS-CoV-2
Source: Nat Biomed Eng. 2023 Nov 23;8(3):291–309. doi: 10.1038/s41551-023-01140-z (PMC10963270; doi:10.1038/s41551-023-01140-z)

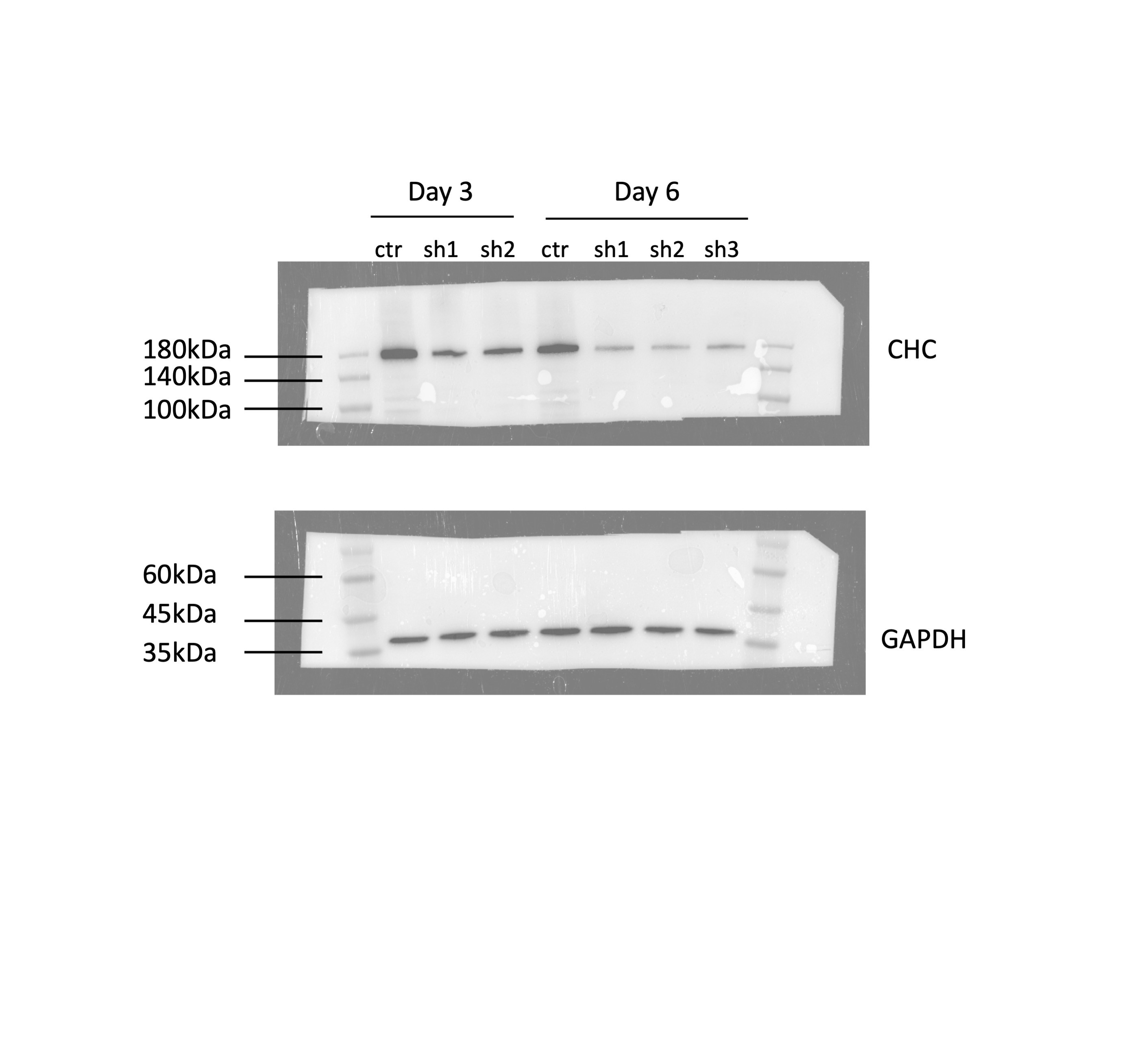

Supplement: Supplementary file 5 — Unprocessed western blots for Fig. 4f. [file 41551_2023_1140_MOESM5_ESM.jpg]
